# Does COVID-19 Spread Through Droplets Alone?

**DOI:** 10.3389/fpubh.2020.00163

**Published:** 2020-04-24

**Authors:** Thushara Galbadage, Brent M. Peterson, Richard S. Gunasekera

**Affiliations:** ^1^Department of Kinesiology and Health Science, Biola University, La Mirada, CA, United States; ^2^Department of Chemistry, Physics, and Engineering, Biola University, La Mirada, CA, United States

**Keywords:** coronavirus, COVID-19, SARS-CoV-2, droplet, viral transmission, pandemic, outbreak, epidemic

## Introduction

The world is in the middle of a historic public health crisis. As of March 30, 2020, over a third of the population in the United States were under “stay at home” orders given by state governors to protect the vulnerable and the unexposed. Unprecedented steps have been taken by governments globally to contain the novel coronavirus disease 2019 (COVID-19), a rapidly spreading pandemic. This has resulted in more than 690,000 cases and over 33,000 deaths worldwide (Supplementary Table 1). The index case of the disease, caused by the Severe Acute Respiratory Syndrome Coronavirus-2 (SARS-CoV-2) was identified more than 3 months ago. Since then, public health authorities worldwide have taken aggressive measures to blunt the exponential spread of this coronavirus. Furthermore, several nations, including Italy, Spain, and France, have imposed nationwide lockdown measures to enforce social distancing to further prevent the spread of COVID-19 in their respective countries.

While preventative measures have been imposed globally, the observed propagation of COVID-19 has noticeable differences among select nations. Epidemiologic data show that some countries have exponential increases in disease incidence, while others seem to have “flattened the curve.” This raises questions of whether a full scientific understanding of disease transmission modes has yet to be attained, and thus whether there are more effective ways to prevent its spread. This brings us to the fundamental question: **Does COVID-19 Spread through Droplets Alone?**

**Figure 1:**
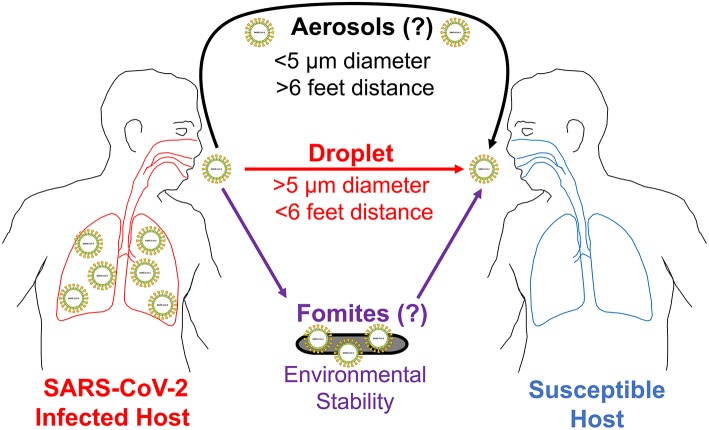
COVID-19 potential modes of transmission. This illustration shows three potential ways SARS-CoV-2 can spread from an infected host to a susceptible host. First, it is transmitted person to person (direct contact) through respiratory droplets. These droplets can travel for distances 6 feet or less in air. Second, SARS-CoV-2 is likely transmitted through fomites (indirect contact) for the duration it is viable on environmental surfaces. Third, it is also likely transmitted through aerosols (indirect contact) for distances longer than 6 feet in the air. To establish an infection, SARS-CoV-2 needs to first reach an entry point (eyes, nose, or mouth) on a susceptible host.

To answer this question, we provide epidemiological observational data in conjunction with known molecular characteristics of SARS-CoV-2. We discuss the ability of this novel coronavirus to remain viable on environmental surfaces from hours to days and describe its increased virulence characteristics compared to the previous SARS-CoV-1. These biochemical and molecular properties likely allow this novel coronavirus to employ indirect methods of transmission, including fomites and aerosols, in addition to respiratory droplet transmission (Figure 1).

Public health measures of this aggressive nature have the universal purpose of reducing the exponential rise in incidence rates of disease transmission. Observations made in health outcomes following the 1918 influenza pandemic have guided public health policy regarding these preventative measures. Importantly, during this pandemic, some U.S. cities chose more effective measures to address the spread of the disease, resulting in observable differences in mortality rates across the nation (1). Social distancing is an evidence-based practice to help prevent the transmission of pathogens that are known to spread from person to person within a 3–6 feet distance through respiratory droplets (2, 3). This practice requires individuals in a community to choose behaviors that increase the physical distance between themselves and others (infected, asymptomatic carriers, or non-infected). Social or physical distancing helps reduce the transmission of respiratory droplets containing SARS-CoV-2 and slows the incidence of the disease by reducing the opportunities for potential viral exposures. Furthermore, this is an excellent example of how integral the public health system and policies are to the proper function of medical and healthcare systems. Acting swiftly and mobilizing precautionary measures can substantially aid in flattening the disease incidence curve, thereby reducing the number of critically ill patients who will need medical treatment all at the same time. This, in turn, reduces the burden on the healthcare system that takes care of patients presenting with the most feared complication of COVID-19, i.e., severe bilateral pneumonia (4). This concept, now widely referred to as flattening the curve, gives critically ill patients a fighting chance to survive by obtaining life-saving supportive therapy in hospitals. This, therefore, significantly reduces the mortality rate (1). If the number of critically ill patients is higher than what can be accommodated in hospitals, many more patients will die due to the lack of life-saving medical attention.

The current consensus regarding the transmission of SARS-CoV-2 is that it spreads person to person through respiratory droplets (5, 6). Precautions to prevent the spread by droplets as recommended by both the Centers for Disease Control and Prevention (CDC) and the World Health Organization (WHO) are to (1) wash hands with soap, (2) avoid touching viral entry points, such as eyes, nose, and mouth, (3) cover the mouth when coughing or sneezing, (4) wear a facemask if sick and (5) practice social distancing by putting 6 feet of distance between individuals. In addition to these precautions, government-mandated social distancing measures such as (6) state lockdowns and (7) “stay at home” orders are effective ways to minimize the spread of SARS-CoV-2 through droplet transmission. Despite all these aggressive precautionary measures, SARS-CoV-2 has succeeded in establishing an exponentially growing pandemic that has spread to almost every nation in the world (Supplementary Figures 1, 2).

## Why Is SARS-CoV-2 Succeeding to Spread in This Trajectory?

Specific epidemiological observations may provide evidence to suspect that the spread of SARS-CoV-2 may not be limited to respiratory droplets alone. For example, on February 4, 2020, the Diamond Princess Cruise ship carrying 3,711 passengers and crew members reported 10 cases testing positive for COVID-19 after their 14-day voyage. As a response to this, the ship was quarantined for 14 days while docked off the coast of Japan. Following this quarantine period, a total of 634 cases reportedly tested positive for COVID-19, despite droplet precautions and social distancing principles practiced on board (7). In retrospect, public health officials acknowledge this was not the best practice implemented to contain COVID-19. Additionally, public health officials responded differently to the Grand Princess Cruise ship off the coast of Oakland, California, based on suspicions that the dramatically widespread transmission of fomites or COVID-19 aerosols may have been exacerbated by interconnected central ventilation between ship cabins (8). Public health officials removed all susceptible and unexposed passengers from this cruise ship, which resulted in a significantly lower number of COVID-19 cases (8).

Tragically, another story that is unfolding in the COVID-19 pandemic is occurring within the country of Italy, which currently maintains a mortality rate of 9.3% (Supplementary Table 1). Once the number of COVID-19 positive cases surpassed 5,000, the government of Italy imposed a nationwide lockdown measure on March 9th (Supplementary Figure 3, solid black arrow). However, even after these measures were in place for over 2 weeks (dotted black arrow), the number of cases of COVID-19 continued to rise exponentially, surpassing 50,000 cases by March 22nd (Supplementary Table 1, Supplementary Figures 2, 3). This may suggest that Italy responded far too late to implement preventative measures that could have flattened the curve. Or, this example may indicate that even amidst the aggressive precautionary measures taken to reduce droplet spread, other modes of transmission may have also occurred. These observations are not limited to just Italy. To date, many of the European nations are experiencing an exponential increase in the incidence rate of COVID-19 despite many stringent precautionary measures employed over the past several weeks (Supplementary Figure 3). These epidemiological observations in the rapid spread of the disease across nations practicing droplet precautions strongly suggest there may be other modes of disease transmission involved (Figure 1).

## What Are Other Modes of Disease Transmission Contributing to the Spread of COVID-19?

Recent studies have indicated that SARS-CoV-2 demonstrated 10–20 times greater affinity to angiotensin-converting enzyme 2 (ACE2) receptors compared to SARS-CoV-1, making it a much more virulent virus (9, 10). This means **fewer SARS-CoV-2 virions are necessary** to establish an infection in humans. This, in part, could explain the rapid spread of the disease worldwide compared to the 2002–2003 SARS outbreak that infected approximately 8,100 individuals.

The primary mode of transmission of SARS-CoV-1 in the 2002–2003 outbreak was by respiratory droplets up to a distance of about 6 feet (3, 11). However, SARS-CoV-1 has also shown to be viable on a variety of common surfaces under environmental conditions up to 96 h post-exposure (12, 13). SARS-CoV-2 was recently shown to remain viable on average for about 6.8 h on plastic surfaces and about 5.6 h on stainless steel surfaces, and viable virions were detected up to 72 h post-exposure (14). These studies have demonstrated that SARS-CoV-2 can **remain viable in the environment much longer** than most other viruses transmitted through respiratory droplets.

The ability of SARS-CoV-2 to remain viable longer on surfaces taken together with its higher virulence in establishing an infection makes it very likely that this coronavirus uses other modes of transmission in addition to respiratory droplets (Figure 1). Remaining longer in the environment may mean this coronavirus can easily transmit through **indirect transmission** routes. This can be either a certain level of airborne spread or vehicle-borne (fomites) transmission. Pathogens like influenza virus and rhinovirus that usually spread through respiratory droplets have some airborne transmission properties making it plausible that SARS-CoV-2 may have such characteristics as well (2, 15, 16). Coughing, sneezing, and talking can produce droplets of various sizes. Fluid mechanical principles show that exhaled droplets smaller than 10 μm can travel longer distances through air streams (17). Respiratory droplets <50 μm can also remain suspended in the air long enough to contaminate ventilation systems located over 12 feet from the source (18). With the ability to remain viable longer in the environment, SARS-CoV-2 likely transmits more than 6 feet in the air.

Such additional modes of transmission can help further explain the observations made on the Diamond Princess Cruise ship in Italy and other European nations. On the cruise ship, contaminated surfaces and utensils (fomites), and aerosolized viral particles traveling beyond 6 feet could have exacerbated the volatile spread of COVID-19. In Italy, having houses or other domiciles close to one another may have transmitted the disease even with a limited level of aerosolization. This example may also better explain the current exponential spread of SARS-CoV-2 in many European nations and in the United States that are aggressively practicing social distancing.

## How Can the Spread of the Coronavirus be Better Prevented?

Today, the world is facing a particularly deadly disease to which there is no cure currently nor a vaccine. Based on the findings mentioned above, if SARS-CoV-2 is also transmitted through indirect contact, additional, yet practical methods of precaution may be indicated. There are ways to help prevent such spread. (1) First, it is essential to follow all droplet precautions including washing hands with soap or using an alcohol-based hand sanitizer for 20–40 s, (2) protecting viral entry points, (3) covering one's mouth when coughing or sneezing, and (4) appropriate social/physical distancing. In addition, (5) continually disinfecting contact surfaces can eliminate the risk of fomite-based transmission. (6) Furthermore, to prevent the possible spread of aerosolized SARS-CoV-2 infections, we will need to reevaluate the current recommendations of 6 feet of physical separation between individuals to possibly increasing it further. Also (7), infected hosts can help prevent the propagation of the virus by donning a face mask covering their mouth and nose to disrupt the airflow near the source. (8) CDC's latest recommendation that all individuals wear a cloth face mask addresses asymptomatic carriers, also known as silent spreaders, and will help protect susceptible hosts. Finally in areas at increased risk of COVID-19 transmission such as hospitals and patient care facilities, (9) appropriately fitted N95 respiratory (facemask), with other personal protective equipment (PPE) and (10) expanded use of special air handling and ventilation systems (e.g., AIIRs) need to be in place (19, 20). This can help contain and safely remove SARS-CoV-2 likely transmitted through aerosolization.

## Author Contributions

TG compiled the epidemiological data, prepared the figures, and helped with the writing and editing of the manuscript. BP discussed the modes of disease spread and helped with the writing and editing of the manuscript. RG discussed the molecular basis of the study and helped with the writing and editing of the manuscript.

## Conflict of Interest

The authors declare that the research was conducted in the absence of any commercial or financial relationships that could be construed as a potential conflict of interest.
