# Supplementary material for: Does COVID-19 Spread Through Droplets Alone?
Source: Front Public Health. 2020 Apr 24;8:163. doi: 10.3389/fpubh.2020.00163 (PMC7193306; doi:10.3389/fpubh.2020.00163)
Supplement: Supplementary file 1 [file Data_Sheet_1.pdf]

# Does COVID-19 Spread through Droplets Alone?

**Thushara Galbadage[1], Brent M. Peterson[1], and Richard S. Gunasekera[2] ***

*[1]Department of Kinesiology and Health Science, [2]Department of Chemistry, Physics, and Engineering, Biola University, La Mirada, California 90639, United States.*

## Supplementary Material

## 1 Supplementary Figures and Tables

### 1.1 Supplementary Figures

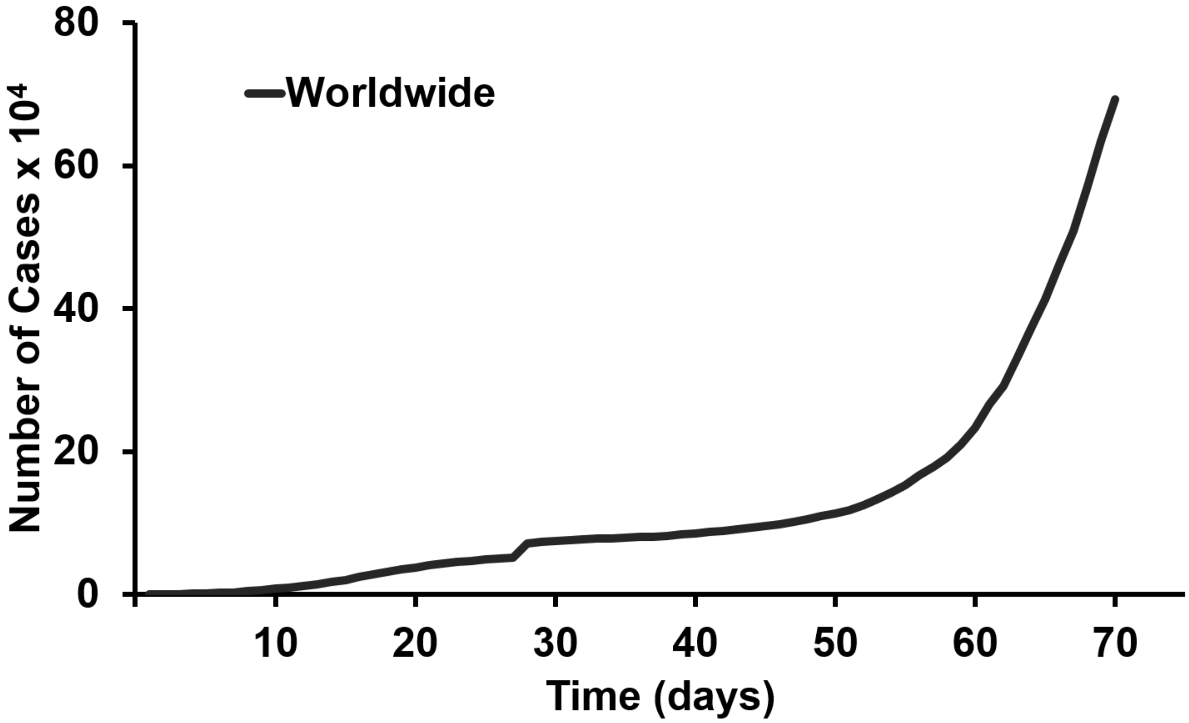


**Supplementary Figure 1. Total Number of Cases of COVID-19 Worldwide.** The cumulative case total of COVID-19 (SARS-CoV-2), as reported by the World Health Organization (WHO) daily COVID-19 Situation Reports (1 – 70). This includes lab-confirmed COVID-19 cases reported from January 21st to March 30th, 2020. As of March 30th, there were 693,224 cases reported worldwide.

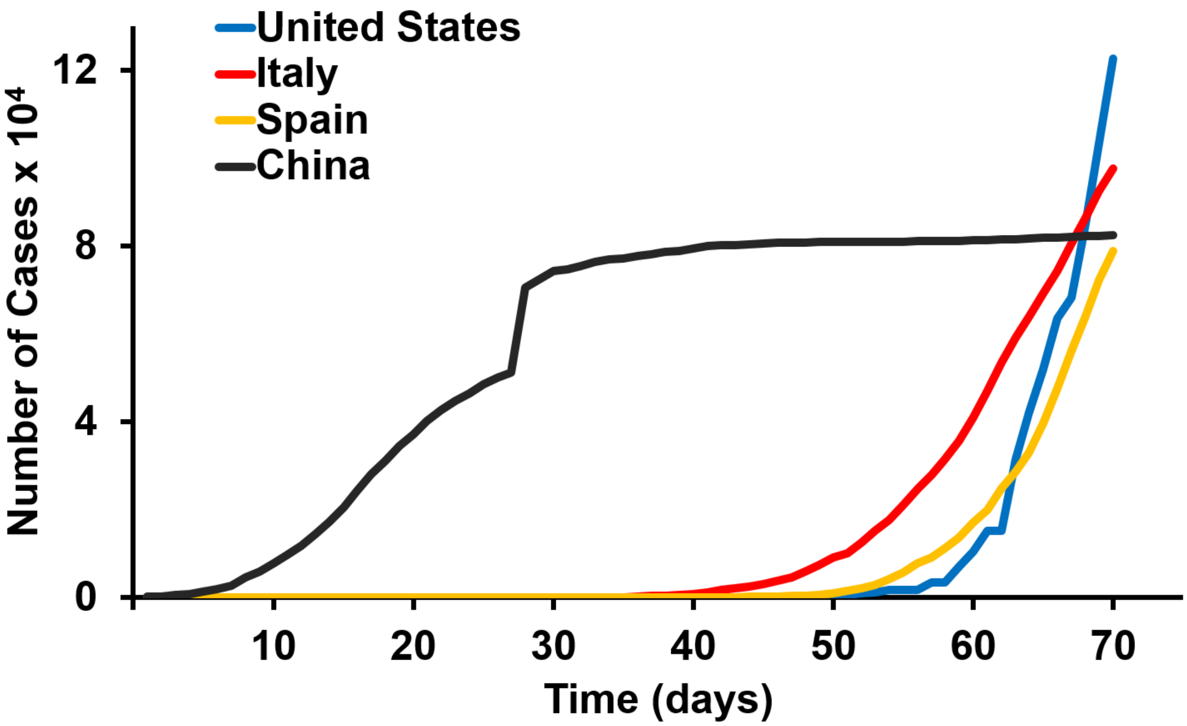


**Supplementary Figure 2. Number of Cases of COVID-19 in the United States, Italy, Spain, and China.** The cumulative case total of COVID-19 (SARS-CoV-2), as reported by the World Health Organization (WHO) daily COVID-19 Situation Reports (1 – 70). This includes lab-confirmed COVID-19 cases reported from January 21st to March 30th, 2020. As of March 30th, there were 122,653 cases in the United States, 97,689 cases in Italy, 78,797 cases in Spain, and 82,447 cases in China. On March 30th, these four countries reported the highest cumulative cases totals of COVID-19 worldwide and represented the countries from North America, Europe, and Asia.

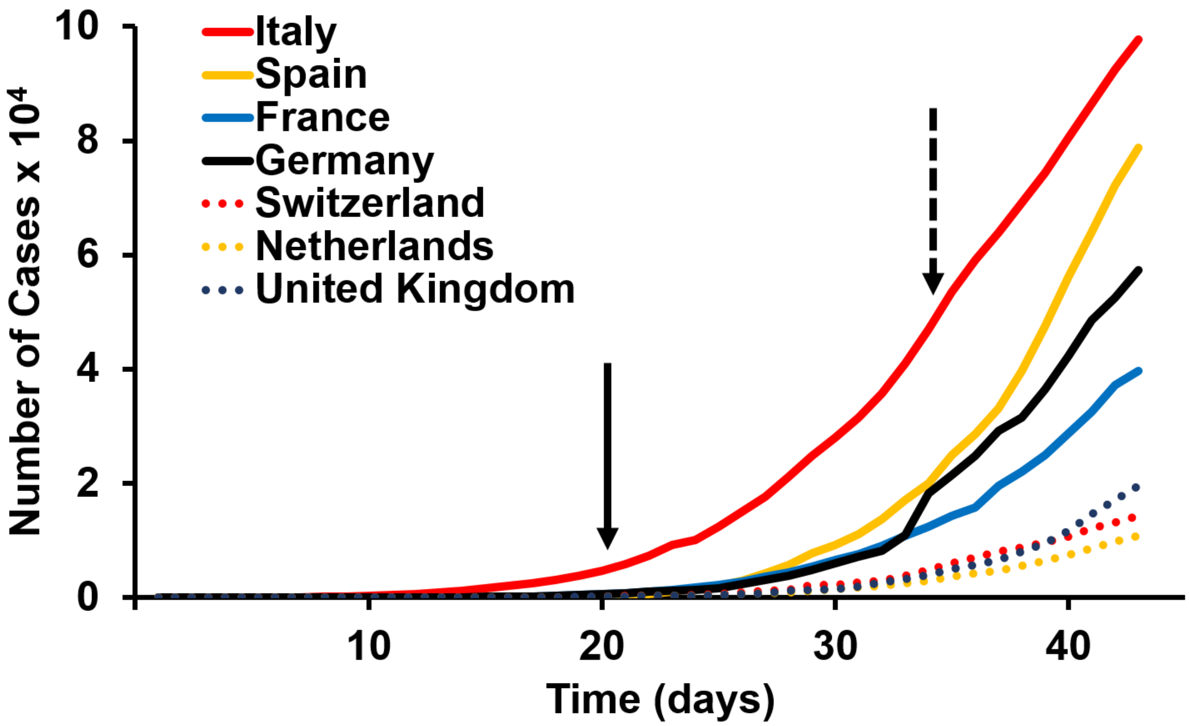


**Supplementary Figure 3. Number of Cases of COVID-19 in Select Countries in the European Region.** The cumulative case total of COVID-19 (SARS-CoV-2), as reported by the World Health Organization (WHO) daily COVID-19 Situation Reports (28 – 70). This includes lab-confirmed COVID-19 cases reported from February 17$^{th}$ to March 30$^{th}$, 2020. As of the March 30$^{th}$, 97,689 cases in Italy, 78,797 cases in Spain, 39,642 cases in France, 57,298 cases in Germany, 14,274 cases in Switzerland, 10,866 cases in Netherlands and 19,526 cases in the United Kingdom were reported. The solid black arrow indicates the 9$^{th}$ of March, 2020, the date Italy entered a nationwide lockdown. The dotted black arrow represents two weeks since the start of the lockdown measures, which overlaps with the incubation period of SARS-CoV-2.

## 1.2 Supplementary Tables

**Supplementary Table 1.** Mortality Rates of COVID-19 as of March 30th, 2020, in Select Regions and Countries.

| Country | Number of Cases[1] | Mortality Rate (%)[2] |
|---|---|---|
| ***Western Pacific Region*** | | |
| China | 82,447 | 4.0 |
| South Korea | 9,661 | 1.6 |
| Japan | 1,866 | 2.9 |
| Malaysia | 2,470 | 1.4 |
| Singapore | 844 | 0.4 |
| Philippines | 1,418 | 5.0 |
| Australia | 3,966 | 0.4 |
| ***European Region*** | | |
| Italy | 97,689 | 11.0 |
| Spain | 78,797 | 8.3 |
| France | 39,642 | 6.6 |
| Germany | 57,298 | 0.8 |
| Switzerland | 14,274 | 1.8 |
| Netherlands | 10,866 | 7.1 |
| United Kingdom | 19,526 | 6.3 |
| ***South-East Asia Region*** | | |
| Thailand | 1,524 | 0.6 |
| India | 1,071 | 2.7 |
| Sri Lanka | 120 | 0.8 |
| ***Eastern Mediterranean Region*** | | |
| Iran | 38,309 | 6.9 |
| United Arab Emirates | 570 | 0.5 |
| ***North American Region*** | | |
| United States of America | 122,653 | 1.7 |
| Canada | 5655 | 1.1 |
| **Worldwide (All Cases Globally)** | **693,224** | **4.8** |

[1] Number of cases of COVID-19 as reported by the World Health Organization (WHO) in their Coronavirus disease 2019 (COVID-19) Situation Report – 70, published on March 30th, 2020.

[2] Mortality rate (%) calculated by taking the ratio of the total number of deaths and the total number of cases reported by the WHO for each country in their COVID-19 Situation Report - 70 published on March 30th, 2020.
